# Combined Genome-Wide and Phenotypic Profiling of *Lactiplantibacillus plantarum* XHQ-007: Genome-Guided Insights into Tyramine Reduction, Safety Characteristics, and Probiotic Potential

**DOI:** 10.3390/foods15111977

**Published:** 2026-06-02

**Authors:** Lang-Hong Wang, Haiqian Xu, Siyu Chen, Weitong Wu, Zhong Han, Xin-An Zeng, Yanyan Huang

**Affiliations:** 1 Guangdong Provincial Key Laboratory of Intelligent Food Manufacturing, School of Food Science and Engineering, Foshan University, Foshan 528225, China; wlhong@fosu.edu.cn (L.-H.W.); 15819320870@163.com (H.X.); 13631435054@163.com (S.C.); 13670617113@139.com (W.W.); huang_yanyan@fosu.edu.cn (Y.H.); 2 School of Food Science and Engineering, South China University of Technology, Guangzhou 510641, China

**Keywords:** *Lactiplantibacillus plantarum*, whole-genome sequencing, tyramine degradation, multicopper oxidase, food safety, probiotic potential

## Abstract

In this study, *Lactiplantibacillus plantarum* XHQ-007, sourced from fermented pineapple pomace, was investigated using whole-genome sequencing (WGS) and systematic phenotypic assays to evaluate its biosafety and functional potential in food applications. The strain showed a 79.94% reduction in detectable tyramine under complex fermentation conditions. In the absence of pH-matched abiotic controls, this reduction cannot be exclusively attributed to enzymatic degradation and may also involve chemical or acid-mediated matrix effects. Further studies are required to distinguish biological degradation from physicochemical contributions. Genomic analysis suggested that this capability may be associated with a putative candidate tyramine-associated metabolic pathway involving the multicopper oxidase *cueO* and the putative *hpa* gene cluster, rather than conventional amine oxidases. Furthermore, the isolate displayed strong resilience against gastrointestinal stressors, such as acidic conditions and bile salts. Safety assessments confirmed the absence of hemolytic activity and mobile antibiotic resistance genes. Notably, WGS also identified a plantaricin (*pln*) operon containing regulatory elements (*plnABCD*) and structural genes (*plnMNOP*), suggesting genomic potential for bacteriocin-related functions. The identification of *poxL* and *nox2* may further indicate energy metabolism and redox homeostasis. Overall, this study provides a genome-informed and phenotype-supported characterization of *L. plantarum* XHQ-007 in a fermentation context. However, all mechanistic interpretations remain putative and require further experimental validation.

## 1. Introduction

Biogenic amines are a class of bioactive nitrogen-containing small-molecule compounds widely found in various fermented foods. When consumed in moderation, they play a role in physiological processes such as nerve transmission and blood pressure regulation; however, if the concentration of biogenic amines in food exceeds the body’s ability to metabolize them via monoamine oxidase, it may trigger severe toxic reactions. Among these, tyramine has a strong vasoconstrictive effect and is often associated with migraines, hypertensive crises, and even cerebral hemorrhage; such adverse reactions are referred to as the “cheese reaction” [1].

With the global growth in consumption of traditional fermented foods, controlling tyramine levels in food has become a research priority in the field of food safety. Existing physicochemical control methods, such as ultra-high-pressure processing or chemical preservatives, have limited effectiveness in inhibiting biamine accumulation and often compromise the characteristic flavors of food, making it difficult to meet current consumer preferences for “clean label” products and natural preservatives. Therefore, the development of biological control agents capable of efficiently degrading tyramine—particularly through biodegradation using proprietary probiotics with this function—has potential value in fermented food control systems [2].

*Lactiplantibacillus plantarum* is a metabolically diverse, environmentally adaptable lactic acid bacterium (LAB) recognized for its probiotic properties and fermentation potential. High-throughput sequencing has advanced genomic research, with whole-genome sequencing enabling comprehensive genetic analysis. This technology systematically evaluates strain safety through virulence factor annotation and antibiotic resistance gene screening, while elucidating probiotic mechanisms at the molecular level [3]. According to the FAO and EFSA guidelines, probiotic safety assessment must cover hemolytic activity, biogenic amine production, and horizontal gene transfer risks. Functional criteria include tolerance to physiological stresses (pH, bile salts), gastrointestinal survival, and transient colonization ability. Furthermore, comparative genomics is essential for understanding intraspecific diversity and functional evolution. Through genomic homology analysis, comparison of metabolic gene clusters, and phylogenetic reconstruction, researchers can map the evolutionary trajectories of adaptation within specific ecological niches [4]. These high-throughput methods support research on tyramine degradation pathways and, through functional annotation and analysis of stress response gene clusters, lay the foundation for designing effective food-grade bioprotection strategies [5].

This study presents *L. plantarum* XHQ-007, a novel strain isolated from fermented pineapple residue in China, which exhibits detectable tyramine reduction under the tested conditions. Through genomic and in vitro analyses, we explored genome-guided hypotheses potentially associated with tyramine reduction and adaptation to a high-acid, high-sugar environment. Safety evaluation per 2025 EFSA guidelines covered antibiotic resistance, hemolysis, and harmful metabolites, alongside assessment of gastrointestinal tolerance and intestinal adhesion. *L. plantarum* XHQ-007 may be considered a candidate strain of potential interest for food fermentation applications for tyramine control in fermented foods and offers a multi-omics reference for functional research on lactic acid bacteria.

## 2. Materials and Methods

### 2.1. Materials and Strains

*Lactiplantibacillus plantarum* XHQ-007 was isolated from fermented pineapple peel residue and deposited at the Guangdong Microbial Culture Collection Center (GDMCC, Guangzhou, China), with the accession number GDMCC No. 66,016. The positive control strains *L. plantarum* DMDL 9010 (GDMCC no. 5172), *E. coli* ATCC 35150 (GDMCC no. 1.707), *E. coli* ATCC 9637 (GDMCC no. 1.355), *S. aureus* ATCC 25923 (GDMCC no. 1.174), *L. monocytogenes* ATCC 19115 (GDMCC no. 1.347), and *S. enteritidis* ATCC 9120 (GDMCC no. 1.1114). The fermented pineapple peel residue samples were sourced from Xuwen County, Zhanjiang City, Guangdong Province, China.

Tyramine hydrochloride was purchased from Shanghai Aladdin Company Limited (Shanghai, China). All other reagents were commercially available analytical grade or biochemical grade. Organic solvents, water, and standards used in high-performance liquid chromatography (HPLC) analysis were chromatographic-grade, while other reagents were analytical grade or biochemical grade.

### 2.2. Screening of LAB with Tyramine Degradation Potential

Following and modifying the method described by Sun [6], strains that had been activated three times were spread onto a double-layer chromogenic medium. After 3 days of incubation, the top layer of chromogenic reagent was added, and the plates were observed for 5 min. A purple color indicated a positive result for biogenic amines, while a yellow color (or no color change) indicated a negative result. Negative strains were selected for subsequent experiments.

The tyramine degradation capacity was evaluated in accordance with the GB 5009.208-2016 standard [7]. Chromatographic conditions included a C18 column (250 mm × 4.6 mm, 5 μm), column temperature of 35 °C, UV detection at 254 nm, mobile phase A consisting of ultrapure water, and mobile phase B consisting of acetonitrile, with a flow rate of 0.8 mL/min and an injection volume of 20 μL. The elution program is shown in Table S1. After activating the screened strains twice, the initial bacterial concentration was adjusted to 1 × 10^9^ CFU/mL and inoculated into MRS medium containing 500 mg/L tyramine. The culture was incubated at 37 °C for 48 h. An abiotic incubation control consisting of tyramine-containing MRS medium without bacterial inoculation was simultaneously incubated under identical conditions to evaluate possible non-biological tyramine loss during the incubation period. To further distinguish biological degradation from possible physicochemical adsorption effects, additional heat-killed and adsorption control experiments were performed. For the heat-killed control, bacterial suspensions were treated at 121 °C for 15 min prior to inoculation into tyramine-containing MRS medium under the same incubation conditions. For the adsorption control, bacterial cells were harvested by centrifugation, washed twice with sterile phosphate-buffered saline (PBS), and resuspended in tyramine solution without nutrients to evaluate passive tyramine adsorption by the cell surface.

After incubation, all samples were centrifuged at 4 °C and 8000 rpm for 5 min, and the supernatants were collected for tyramine quantification by high-performance liquid chromatography (HPLC). Strains with strong tyramine degradation capacity were selected as target strains and stored at −80 °C (in 25% glycerol). The formula for calculating the tyramine degradation rate is as follows:

Degradation rate of biogenic amines (%) = (A − B)/A × 100(1)

where A denotes the tyramine concentration in the initial solution (mg/L), and B denotes the residual tyramine concentration after the degradation process (mg/L).

One milliliter of the sample solution was accurately pipetted into a 10 mL centrifuge tube, and the following added in sequence: 0.20 mL of 2.00 M NaOH solution to make the solution alkaline, 0.30 mL of saturated Na_2_CO_3_ solution to buffer the solution, and 2.00 mL of dansulfonyl chloride derivative solution (10.00 mg/mL, dissolved in acetone); it was vortexed for 60 s, then reacted in a 40 °C constant-temperature water bath away from light for 45 min, removing the tube every 15 min to vortex for 60 s. The reaction was stopped by adding 100 μL of 100% ammonia solution, then let stand in the dark at 25 °C for 30 min. It was then diluted to 5.00 mL with acetonitrile. The mixture was centrifuged at 4 °C and 5500 rpm for 5 min, then the supernatant filtered through a 0.22 μm filter membrane. The tyramine concentration was determined in the supernatant using HPLC (Agilent 1260, Agilent Technologies, Santa Clara, CA, USA).

### 2.3. Screening for Acid- and Bile Salt-Resistant Strains

The activated strains were inoculated at a 2% (*v*/*v*) inoculum volume into MRS medium adjusted to pH 2.0, 4.0, and 6.0 using HCl or NaOH, as well as into MRS medium containing bile salt concentrations of 0.1%, 0.3%, 0.5%, and 1.0%. Cultures were incubated anaerobically at 37 °C for 24 h. Live bacterial counts were determined on MRS agar plates using the serial dilution plate count method. Survival rate was calculated as follows:

Survival (%) = N_t_/N_0_ × 100 (2)

where N_0_ and N_t_ represent colony forming units (CFU) formed before and after acid treatment, respectively.

### 2.4. Strain Identification and Characterization

#### 2.4.1. Morphological Observation

The activated strain was inoculated into MRS liquid medium and incubated overnight at 37 °C. Subsequently, the bacterial suspension was spread onto MRS solid medium to observe colony morphology. Simultaneously, following the method described by Zhang [8], the microscopic morphology of the strains was examined using a scanning electron microscope (Quattro S, Thermo Fisher Scientific, Waltham, MA, USA). Additionally, the activated bacterial suspension underwent Gram staining, and the staining results were observed under an optical microscope (Mshot MF53, Guangzhou Micro-shot Technology Co., Guangzhou, China).

#### 2.4.2. Physiological and Biochemical Assays

The physiological and biochemical characteristics of the screened strains were determined using glucose phosphate peptone water (acetomethyl methanol, methyl red test), hydrogen sulfide, carbohydrate fermentation tubes, gelatin medium, and starch medium. Carbon source utilization capacity was assessed using the API 50 CHL kit (HuanKai Microbiology, Guangzhou, China) to identify the screened strains. Specifically, fresh bacterial cultures were inoculated onto test strips containing 49 carbon source substrates. Cultures were incubated at 37 °C for 24 and 48 h, with results recorded and analyzed using the API bacterial identification system.

#### 2.4.3. Identification of Strain 16S rDNA

DNA was extracted from LPXHQ-007 and amplified via PCR using universal bacterial 16S rDNA primers. Amplified products were sequenced, and results were uploaded to the NCBI database for sequence homology comparison. Subsequently, 16S rDNA sequences of closely related species were downloaded from the NCBI database to construct a phylogenetic tree using MEGA 11.0.10 (LLC, University Park, PA, USA) software.

#### 2.4.4. Determination of Growth and Acid Production Curves

An activated strain was inoculated at a 2% ratio into 100 mL of MRS medium and incubated at 37 °C. Every 2 h, 5 mL of fermentation broth was sampled. Absorbance values were measured at 600 nm against a blank liquid medium control. Simultaneously, the pH of the fermentation broth was monitored.

### 2.5. Whole-Genome Sequencing and Functional Analysis

The genome of LPXHQ-007 strain was sequenced using the PacBio RS II sequencing platform and the Illumina HiSeq 4000 sequencing platform (BGI, Shenzhen, China). Comprehensive gene analysis was performed using COG (COG), GO (Gene Ontology), KEGG (Kyoto Encyclopedia of Genes and Genomes), NR (Non-Redundant Protein Database), and T3SS (Type III Secretion System Effector Protein) databases. Antibiotic resistance genes and virulence factors of LPXHQ-007 were identified using the VFDB (Virulence Factors Database), CARD (Comprehensive Antibiotic Resistant Database), and ARDB (Antibiotic Resistance Database Core).

### 2.6. Comparative Genomic Analysis

The LPXHQ-007 genome sequence was compared with reference strains: *L. plantarum* WCFS1 (NCBI accession: NC_004567); *L. plantarum* ZJ316 (NCBI accession: CP004082); *L. plantarum* JDM1 (NCBI accession: NC_012984); *L. plantarum* 16 (NCBI accession: NC_021514); *L. plantarum* NCU116 (NCBI accession number: NZ_CP016071). Structural differences were detected using MUMmer software (v4.0.0) (http://mummer.sourceforge.net/, accessed on 7 November 2025) to perform comparative genomic analysis, elucidating the relationship between mutations and strain evolution.

### 2.7. Gene Annotation Related to Tyramine Degradation

Genes associated with tyramine reduction potential were annotated using COG and KEGG databases. By integrating KEGG pathway maps and gene information, map the tyramine metabolic pathway in the LPXHQ-007 strain.

### 2.8. Safety Analysis of Strains and Annotation of Related Genes

#### 2.8.1. Antibiotic Resistance Testing

Following the method of Tang et al. with slight modifications [9], the minimum inhibitory concentration (MIC) of antibiotics against strain XHQ-007 was determined using the broth microdilution method. Briefly, an activated bacterial culture was adjusted to approximately 1 × 10^6^ CFU/mL and inoculated into sterile 96-well microplates containing MRS broth supplemented with two-fold serial dilutions of antibiotics. The antibiotics tested included ampicillin, amoxicillin, chloramphenicol, erythromycin, tetracycline, streptomycin, kanamycin, vancomycin, norfloxacin, and gentamicin. After incubation at 37 °C for 24 h, the MIC value was defined as the lowest antibiotic concentration at which no visible bacterial growth was observed.

Antibiotic resistance-related genes were annotated by comparison against the Comprehensive Antibiotic Resistance Database (CARD). A threshold-based filtering strategy was applied, retaining only hits with sequence identity ≥80% and coverage ≥70% for downstream analysis. All matches below these thresholds were excluded from the primary resistance gene profile and considered low-confidence hits.

Mobile genetic elements (MGEs) were analyzed to evaluate the genomic context of antibiotic resistance-related genes. Plasmid-associated sequences were identified using PlasmidFinder, while insertion sequences and transposase-associated regions were screened using ISfinder-based annotation. Prophage regions were predicted using PHASTER. In addition, genomic regions flanking antibiotic resistance-related loci were extracted (±10–20 kb) and examined for the presence of mobile element-associated features, including integrases, transposases, insertion sequences, and plasmid replication proteins.

#### 2.8.2. Indole Test

LPXHQ-007 was inoculated at 2% (*v*/*v*) into indigo base detection medium and incubated at 37 °C for 72 h. A small amount of diethyl ether was added, and the medium was shaken to allow the ether layer to float on the surface. Then, 8–10 drops of Kovacs reagent were added. A red coloration on the surface of the indigo base medium indicated a positive result. LP9010 and *Escherichia coli* ATCC 9637 (EC9637) served as control strains.

#### 2.8.3. Nitroreductase Assay

A nitroreductase assay medium was prepared according to the established method [9]. LPXHQ-007 culture was inoculated and incubated at 37 °C for 48 h. Subsequently, the fermentation broth was mixed with α-naphthylamine solution and p-aminobenzenesulfonic acid solution to observe the resulting color change. EC9637 served as the positive control, while LP9010 acted as the negative control.

#### 2.8.4. Hemolysis Test

The hemolytic activity of LPXHQ-007 was evaluated using a previously established method [10]. Bacterial suspensions were inoculated onto Columbia blood agar plates containing sterile hemolysis rings and incubate at 37 °C for 48 h. Colony characteristics were observed and recorded. LP9010 and *Staphylococcus aureus* ATCC 25923 (SE25923) served as the control strains.

### 2.9. Probiotic Analysis of Strains and Annotation of Related Genes

#### 2.9.1. Tolerance to Artificial Gastric and Intestinal Fluids

Artificial gastric and intestinal fluids were prepared according to the protocol described by Lu [11]. Inoculate activated LPXHQ-007 and LP9010 into simulated gastric fluid (pH 3.0, containing 0.3% pepsin) and simulated intestinal fluid (pH 8.0, containing 0.1% trypsin and 0.3% bile salts), respectively, to achieve a final concentration of approximately 10^9^ CFU/mL. Samples were taken at 0, 30, 60, 90, and 120 min (for gastric and intestinal fluids, respectively) during incubation at 37 °C for viable cell counting.

#### 2.9.2. Antioxidant Assays

The antioxidant activities of LPXHQ-007 and LP9010 were investigated, including hydroxyl radical scavenging activity, superoxide anion scavenging activity, 2,2′-azino-bis (3-ethylbenzothiazoline-6-sulfonic acid) (ABTS) radical scavenging activity, and 2,2-diphenyl-1-picrylhydrazyl (DPPH) radical scavenging activity [12,13].

#### 2.9.3. Coagulation Capacity Assay

LPXHQ-007 strain was mixed with equal volumes of each pathogenic strain (*Escherichia coli* O157:H7 ATCC 35150 (EC35150), *Listeria monocytogenes* ATCC 19115 (LM19115), *Salmonella enterica* ATCC 9120 (SE9120), and *Staphylococcus aureus* ATCC 25923 (SA25923)), with 1.5 mL of each pathogen. After gentle shaking for 90 s, the mixtures were incubated at 37 °C for 1 h and 4 h. Following incubation, measure the absorbance at 600 nm to assess bacterial growth kinetics. LP9010 strain served as the control group.

#### 2.9.4. Cell Surface Hydrophobicity Assay

Surface hydrophobicity was determined according to the method described in Lu [11]. A 3 mL activated bacterial solution was mixed with 1 mL xylene and incubated at room temperature for 10 min. The mixture was then gently shaken for 3 min and left to stand in a well-ventilated area for 30 min to achieve stratification and absorption of the lower aqueous phase. Finally, the absorbance was measured at 600 nm.

#### 2.9.5. Self-Coagulation Rate Assay

Following the method described by Liu [12], the self-coagulation ate of LPXHQ-007 and LP9010 were determined. The absorbance of LPXHQ-007 at 600 nm was adjusted to 0.6, and the culture medium was then incubated at 37 °C for 2, 4, 6, 8, 10, 12, and 24 h. The absorbance of the bacterial supernatant at 600 nm was measured at each time point. LP9010 was used as the control strain. Concurrently, adhesion-related genes were annotated through multi-database analysis (IPR/SWISSPROT/COG/GO/KEGG/NR/T3SS).

#### 2.9.6. Antimicrobial Assay

The antibacterial effects of LPXHQ-007 supernatant against four pathogenic strains were evaluated by agar well diffusion [12]. Indicator strains were mixed with LB agar, and use a sterilized puncher to make holes on the medium. After 24 h incubation at 37 °C, inhibition zones were measured, with LP9010 as control and sterile water as blank. Secondary metabolite and bacteriocin gene clusters in LPXHQ-007 were annotated using antiSMASH and BAGEL4, respectively.

### 2.10. Statistical Analysis

Data were statistically analyzed using SPSS 26.0. For each experiment conducted in triplicate, one-way analysis of variance (ANOVA) and Duncan’s multiple range test were used to evaluate experimental differences at the 0.05 level.

## 3. Results and Discussion

### 3.1. Analysis and Screening of Strains Most Likely to Reduce Tyramine

**Figure 1:**
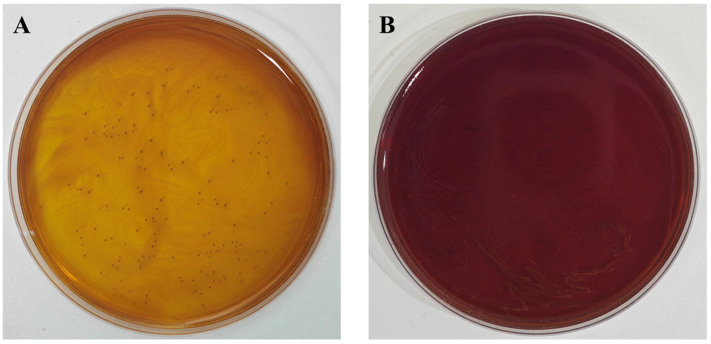
Biogenic amine color reaction ((**A**) indicates negative results for non-ammonia-producing bacteria; (**B**) indicates positive results for ammonia-producing bacteria).

Sixty LAB strains were preliminarily isolated from fermented pineapple peel residue. Using double-layer chromogenic media for screening, strains exhibiting amine production were identified based on color changes (Figure 1), ultimately yielding 46 non-amine-producing strains.

**Figure 2:**
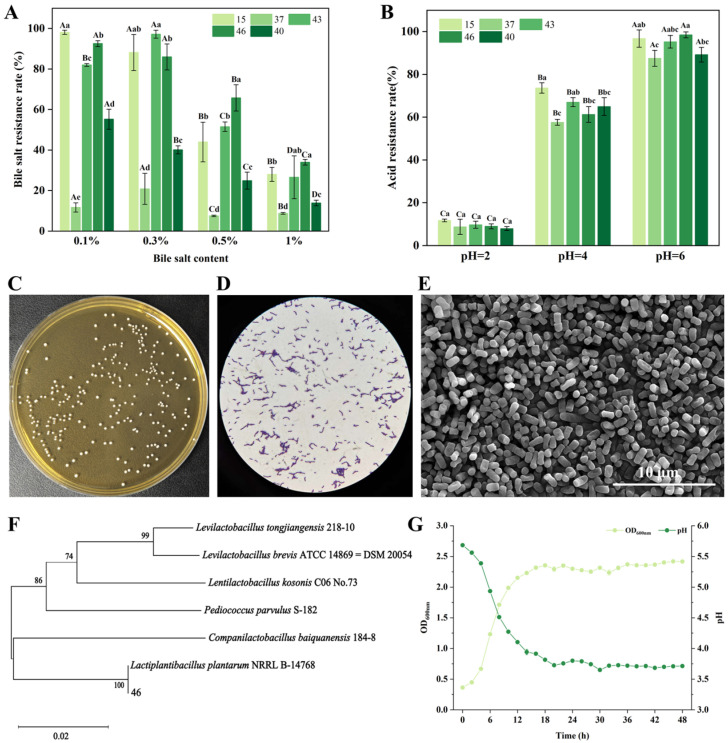
Analysis of tolerance, morphology, phylogeny, and growth characteristics of candidate *Lactiplantibacillus plantarum strains*. (**A**) Represents bile salt tolerance rate; (**B**) represents acid tolerance; (**C**) is the colony morphology of LPXHQ-007; (**D**,**E**) are the bacterial morphologies observed by OM and SEM; (**F**) is the phylogenetic tree and (**G**) is the growth and acid production curve of LPXHQ-007. Data are presented as the mean ± standard deviation (error bars). Different lowercase letters (a, b, c, d, e) above the bars indicate statistically significant differences between the experimental group and the control group for the same treatment (*p* < 0.05). Different uppercase letters (A, B, C, D) above the bars indicate statistically significant differences among different treatment groups (*p* < 0.05).

The tyramine reduction capacity of these 46 strains was further assessed via HPLC. From these, the five strains with the highest degradation rates were selected for evaluation of acid and bile salt tolerance, with results shown in Figure 2A,B. Figure S1 shows the HPLC chromatograms of tyramine reduction for the top 5 strains, and Figure S2 shows the tyramine calibration curve. The tyramine calibration curve showed good linearity within the tested concentration range, with the regression equation y = 31.816x + 62.523 (R^2^ > 0.99). Based on the standard deviation of replicate low-concentration measurements and the slope of the calibration curve, the estimated limit of detection (LOD) and limit of quantification (LOQ) were 0.49 mg/L and 1.50 mg/L, respectively. The recovery rate of tyramine was 92.19%, indicating acceptable analytical accuracy and reliability of the HPLC method under the present experimental conditions. In addition, the intra-assay precision of the method, evaluated using replicate measurements of low-concentration tyramine standards, yielded an RSD value of 2.14%, demonstrating good analytical repeatability. Comprehensive comparison revealed that strain 46 exhibited a 79.94% reduction in detectable tyramine under complex fermentation conditions, together with relatively strong acid and bile salt tolerance. Table S2 shows the tyramine reduction levels of the top ten LAB strains. Strain 46 was selected for further characterization due to its comparatively higher performance across the evaluated phenotypic traits. Under the same culture conditions, the non-biological control group—composed of tyramine-containing MRS medium without bacterial inoculum—exhibited a tyramine loss of only 2.35%, which is negligible, indicating that spontaneous non-biological degradation is very limited. To further distinguish biological degradation from potential physicochemical adsorption effects, heat-killed cell and adsorption control experiments were additionally performed (Figure S3). The heat-killed cells exhibited only limited tyramine reduction compared with viable strain 46 cells, indicating that passive adsorption alone could not fully account for the observed decrease in tyramine concentration. This reduction level is notable when compared with previously reported LAB strains. For example, in a study of 26 *Lactiplantibacillus plantarum* isolates from wine, only 26.9% of the strains degraded tyramine, and the best-performing strain, *L. plantarum* NDT09, showed 22.12% tyramine reduction after 24 h in MRS broth supplemented with tyramine (1 mM) [14]. In another study, the strongest tyramine-degrading activity was observed in *L. casei* CRL705 and CRL678, which reached 98% and 93% degradation, respectively, whereas *L. plantarum* CRL681 and CRL682 showed 69% and 60% degradation, respectively (initial tyramine concentration: 2.5 mM) [15].

Taken together, these findings suggest that the tyramine-degrading capacity exhibited by strain 46 is comparable to values reported for certain previously described LAB strains. However, in the absence of a pH-matched non-biological control, this value reflects the total reduction in detectable tyramine under complex fermentation conditions, and cannot be exclusively attributed to enzymatic degradation, with potential contributions from physicochemical and matrix-associated effects not fully excluded.

### 3.2. Identification and Fermentation Characterization of Strain LPXHQ-007

As shown in Figure 2C, LPXHQ-007 colonies grown on MRS agar were circular and creamy white, with a diameter of approximately 1–2 mm, and exhibited a smooth, moist surface. SEM observation revealed LPXHQ-007 cells as short rod-shaped, occurring singly or in chains (Figure 2D). Morphological analysis confirmed that the colony and cellular characteristics of LPXHQ-007 align with those typical of LAB (Figure 2E).

The 16S rRNA sequence data of strain 46 were analyzed using BLAST (v2.13.0), and a phylogenetic tree was constructed based on these results. As shown in Figure 2F, strain 46 exhibits a high degree of similarity to *L. plantarum* NRRL B-14768 within a specific clade. Therefore, this strain was ultimately named *L. plantarum* XHQ-007.

Average nucleotide identity (ANI) was calculated between LPXHQ-007 and closely related Lactiplantibacillus species, including *L. plantarum*, *L. pentosus*, and *L. paraplantarum*, using FastANI. Genome-wide pairwise ANI values and alignment coverage were calculated to assess taxonomic relatedness. The results were summarized in a comparative Table 1 for clarity.

Microbiochemical reaction tubes were employed to identify LAB and related bacteria, characterizing their physiological and biochemical properties. As shown in the Table 2, LPXHQ-007 exhibited negative results in the hydrogen sulfide test, starch hydrolysis, gelatin liquefaction, and VP tests, while yielding positive results in the MR test and sugar fermentation tests. Based on the aforementioned physiological and biochemical characteristics, LPXHQ-007 demonstrates a high degree of similarity to *Lactiplantibacillus plantarum*.

Growth curve analysis indicates that LPXHQ-007 exhibits typical growth phases under fermentation conditions (Figure 2G). During the exponential growth phase (2–12 h), the strain proliferated rapidly, accompanied by a significant decrease in culture medium pH, which dropped to approximately 4.10 by 12 h, demonstrating strong acid-producing activity. After 12 h, due to the depletion of nutrients and the accumulation of acidic metabolic byproducts, bacterial growth entered a stationary phase. These results demonstrate that LPXHQ-007 possesses outstanding acid-producing capacity and rapid growth characteristics, providing important evidence for its application as a starter strain in food fermentation.

### 3.3. Whole-Genome Sequencing and Analysis of LPXHQ-007

**Figure 3:**
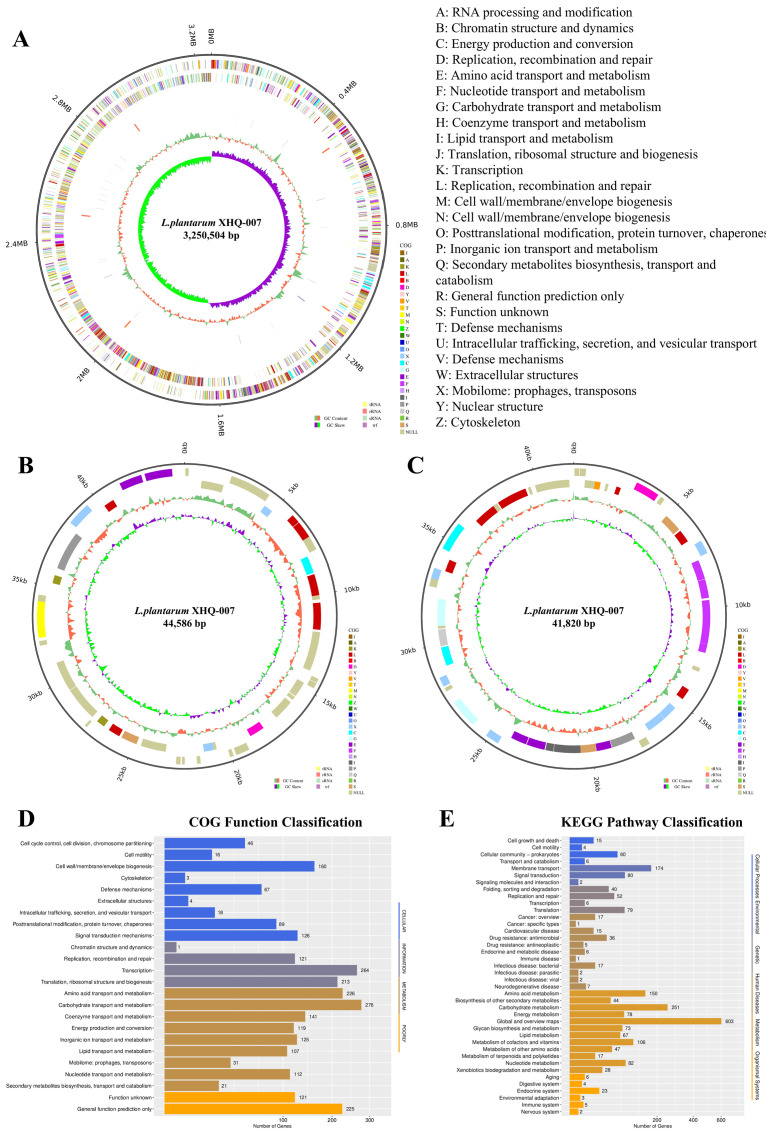
Genome profile of LPXHQ-007 and functional annotations in different databases. (**A**) is the chromosome map of LPXHQ-007; (**B**) is the plasmid 1 ring map; (**C**) is the plasmid 2 ring map; (**D**) is the COG functional classification, and (**E**) is the KEGG functional classification.

LPXHQ-007 possesses one chromosome and two plasmids, with a genome length of 3,336,910 bp. It contains 3206 genes, accounting for 84.05% of the genome, with a cumulative gene length of 2,804,658 bp. The average GC content of genes is 45.56%. The XHQ-007 genome contains 2565 genes annotated in the COG database, including 529 genes involved in cellular composition, 599 genes involved in signal transduction, and 1158 genes involved in metabolism, among others (Figure 3D).

Functional annotation against the KEGG database revealed the core metabolic characteristics of the target genome, with 2216 annotated genes classified into six functional categories of metabolic pathways (Figure 3E). Specifically, 1546 genes are involved in metabolism, 256 in environmental signal processing, 177 in genetic information processing, and 85 in cellular processes. Additionally, 109 and 43 genes are associated with human diseases and organism systems, respectively. Notably, both COG and KEGG databases revealed substantial gene expression regulation annotations involving carbohydrate degradation (276 genes) and synthesis (251 genes), highlighting their impact on metabolic activities. Furthermore, carbohydrate metabolism and membrane transport emerged as the most abundant gene categories observed in this study, indicating the strain possesses robust capabilities for sugar transport and metabolism.

### 3.4. Comparative Genomic Analysis of LPXHQ-007 and Five Other Lactiplantibacillus plantarum Strains

**Figure 4:**
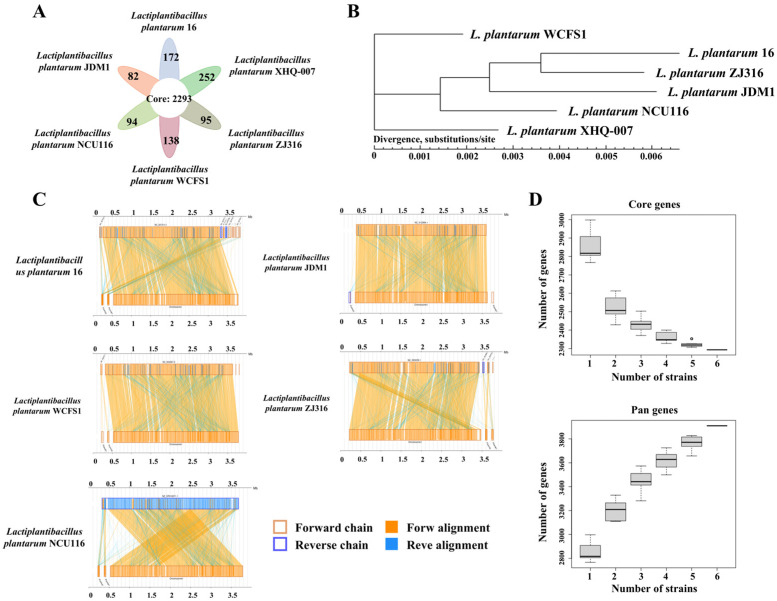
Comparative analysis of LPXHQ-007 and the other five *L. plantarum* strains. (**A**) Represents the core genes and unique genes of the six strains; (**B**) the phylogenetic tree based on the sequence similarity of 2293 core genes; (**C**) the co-linearity analysis of LPXHQ-007 and the other five *L. plantarum* strains at the nucleic acid level. (**D**) The dilution curve of the core genes and pan-genes of LPXHQ-007.

A screening of strains highly similar to the LPXHQ-007 genome in the NCBI database revealed that it shares high genetic similarity with *L. plantarum* 16, *L. plantarum* JDM1, *L. plantarum* NCU116, *L. plantarum* WCFS1, and *L. plantarum* ZJ316, consistent with previous reports. The core genomes of these six strains contain 2293 core genes, while their pan-genomes contain 3909 genes; among them, LPXHQ-007 possesses the highest number of unique genes (252) (Figure 4A). A phylogenetic tree constructed based on core genes indicates that LPXHQ-007 is most closely related to *L. plantarum* WCFS1 (Figure 4B), suggesting that the two share similar physiological and biochemical characteristics.

Genomic synteny analysis helps elucidate variations between sequenced genomes and reference genomes caused by mechanisms such as transposition and inversion. The results indicate that XHQ-007 exhibits high colinearity with *L. plantarum* 16, *L. plantarum* JDM1, *L. plantarum* WCFS1, and *L. plantarum* ZJ316, but contains a localized gene inversion relative to NCU116 (Figure 4C). These structural variations may originate from recombination and the movement of genetic elements during the process of strain differentiation.

Dilution curves (Figure 4D) show core gene numbers decline with fewer genomes but stabilize after six, indicating these six genomes sufficiently cover the species core genome. Conversely, pan-genes continue rising without saturation, revealing LPXHQ-007′s “open” pan-genome structure, reflecting significant genetic diversity and ongoing potential for new gene acquisition.

Although comparative genomic analysis alone cannot establish a direct causal relationship between genotype and phenotype, several genomic characteristics identified in LPXHQ-007 may be associated with the observed tyramine reduction phenotype. Compared with related strains, LPXHQ-007 harbored multiple genes potentially associated with oxidative metabolism, aromatic compound transformation, and intracellular redox regulation, including *cueO*, *hpa*-related genes, *nox2*, and catalase-associated genes. These genomic features may be associated with the observed phenotype to tolerate oxidative stress and metabolize tyramine-derived intermediates under complex fermentation conditions.

### 3.5. Putative Genetic Basis for Tyramine Degradation in LPXHQ-007

In prokaryotes, tyramine degradation typically involves amine oxidase-mediated oxidative deamination to 4-hydroxyphenylacetaldehyde (generating H_2_O_2_ and NH_3_), followed by aldehyde dehydrogenase oxidation to 4-hydroxyphenylacetic acid. This intermediate then enters either the phenylacetic acid degradation pathway or the tricarboxylic acid (TCA) cycle via side pathways.

**Figure 5:**
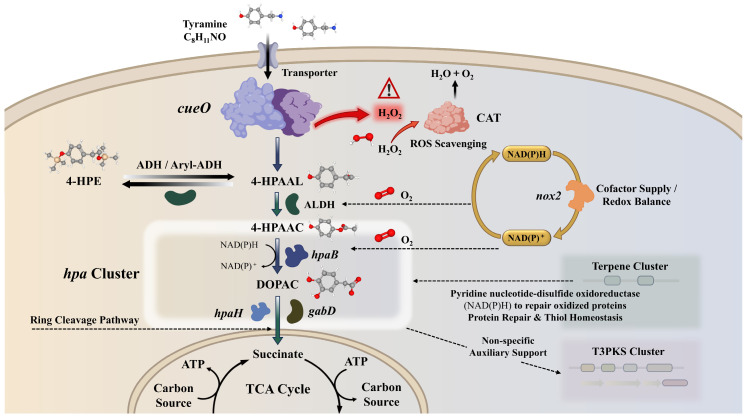
Putative tyramine degradation pathway in LPXHQ-007, inferred from genome annotation and functional gene prediction. *cueO*: multicopper oxidase; ADH: alcohol dehydrogenase; Aryl-ADH: aryl-alcohol dehydrogenase; 4-HDE: 4-Hydroxyphenylethanol; 4-HPAAL: 4-Hydroxyphenylacetaldehyde; 4-HPAAC: 4-Hydroxyphenylacetate; ALDH: aldehyde dehydrogenase; *hpaB*: 4-hydroxyphenylacetate 3-monooxygenase B; DOPAC: 3,4-Dihydroxyphenylacetate; *gabD*: succinate-semialdehyde dehydrogenase; *hpaH*: hydratase; *nox2*: nicotinamide adenine dinucleotide oxidase; CAT: catalase; T3PKS Cluster: type III polyketide synthase cluster; NAD(P)^+^: nicotinamide adenine dinucleotide phosphate; NAD(P)H: nicotinamide adenine dinucleotide phosphate; TCA Cycle: tricarboxylic acid cycle; ATP: adenosine triphosphate.

As shown in Figure 5, although the classical tyramine oxidase is absent from the LPXHQ-007 genome annotation, functional genomic analysis suggested the presence of a putative and non-canonical downstream degradation pathway. First, the cuproxidase gene (*cueO*) was identified in the LPXHQ-007 genome. In recent years, numerous researchers have demonstrated that multicopper oxidases (MCOs) in lactic acid bacteria may exhibit laccase-like activity and could potentially contribute to the oxidative transformation of biogenic amines. The multicopper oxidase (MCOs), centered around *cueO*, exhibits laccase-like activity. Utilizing its four copper centers to mediate electron transfer chains, it may facilitate the conversion of tyramine into the intermediate product 4-hydroxyphenylacetaldehyde via a putative oxidative deamination-like reaction, through oxidative deamination [16]. Subsequently, aryl-alcohol dehydrogenaseand alcohol dehydrogenase can mediate the interconversion between 4-hydroxyphenylacetaldehyde and 4-hydroxyphenylethanol, or be further oxidized by aldehyde dehydrogenase to the key intermediate 4-hydroxyphenylacetate. These genomic features may be consistent with a possible non-canonical oxidation–dehydrogenation route. However, its actual involvement in tyramine metabolism remains uncertain in the absence of functional enzymatic and cellular validation.

More notably, the LPXHQ-007 genome harbors a complete hpa gene cluster, including 4-hydroxyphenylacetate 3-monooxygenase B (*hpaB*), 2-oxo-hept-3-ene-1,7-dioate hydratase (*hpaH*), and succinate-semialdehyde dehydrogenase (*gabD*). The metabolic network diagram reveals that 4-hydroxyphenylacetate is converted to 3,4-dihydroxyphenylacetate under the catalysis of *hpaB*. Subsequently, through a series of cyclolytic reactions, it ultimately generates succinate via the synergistic action of *hpaH* and *gabD*, entering the TCA cycle [17,18]. The presence of these annotated genes suggests that XHQ-007 are consistent with possible downstream metabolism of tyramine-derived intermediates. However, the actual metabolic flux and mineralization process remain to be experimentally validated, indicating a putative catabolic capacity rather than confirmed pathway activity. This genomic organization indicates a potential adaptive advantage for utilization of aromatic amine-derived intermediates in complex fermentation environments.

Additionally, multiple redox-associated genes were identified in the genome, such as catalase (CAT) and NADH oxidase (*nox2*). Among these, CAT provides an efficient scavenging mechanism for oxidative stress (e.g., H_2_O_2_) generated during tyramine oxidation, thereby maintaining intracellular redox balance [19]. The significance of *nox2* lies in regulating the intracellular NAD(P)+/NAD(P)H balance, providing the necessary oxidized cofactor environment for downstream aldehyde dehydrogenase or monooxygenase (*hpaB*) [20]. Additionally, the presence of histidinol–phosphate aminotransferase suggests that this strain may possess transaminase potential across substrates, although its direct involvement in tyramine metabolism remains speculative [21].

**Figure 6:**
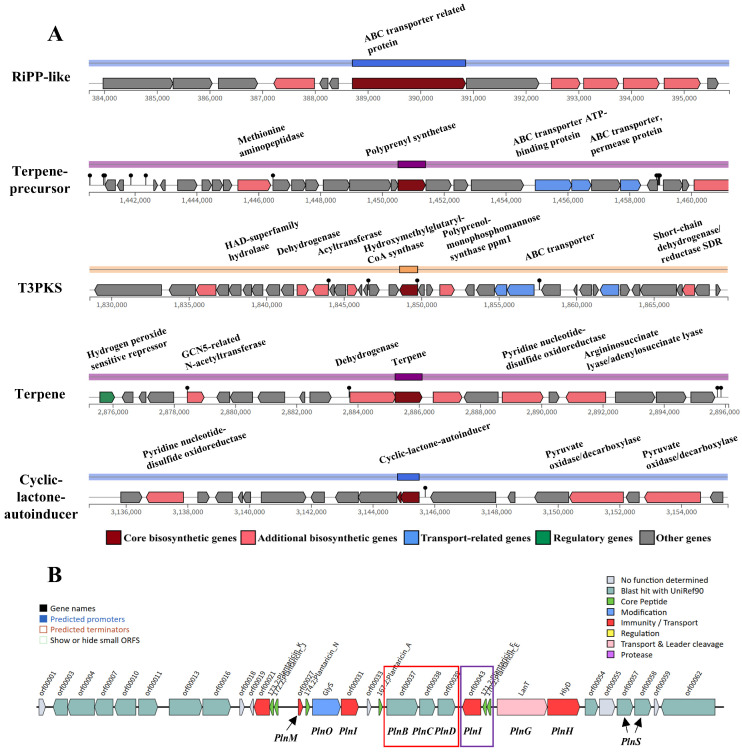
Predicted secondary metabolites of LPXHQ-007. (**A**) Gene clusters annotated by AntiSMASH 7.1.0, and (**B**) represents gene clusters annotated by BAGEL4.

In addition to the functional genes associated with amine reduction mentioned above, AntiSMASH 7.0 analysis identified multiple gene clusters in LPXHQ-007 (Figure 6A). Although these clusters primarily involve secondary metabolite synthesis, the dehydrogenase and acyltransferase identified by the type III polyketide synthase (T3PKS) may play non-specific roles in assisting biogenic amine conversion. This spatial clustering of metabolic functions may aid cells in efficiently processing oxidative stress byproducts (e.g., H_2_O_2_ processed by *catA*) during tyramine degradation. Notably, a pyridine nucleotide-disulfide oxidoreductase was also identified within the cyclic lactone autoinducer and terpene gene clusters. This enzyme typically participates in intracellular disulfide bond reduction and thiol homeostasis. We hypothesize that this enzyme may form a potential functional association with *nox2*-mediated redox regulation: under tyramine degradation conditions generating substantial oxidative stress byproducts, the pyridine nucleotide–disulfide oxidoreductase could repair ROS-oxidized protein disulfide bonds, while *nox2* provides the necessary redox gradient for these repair reactions by regulating cofactor pools [22,23]. This coupling mechanism remains speculative but may be associated with oxidative stress response processes in LPXHQ-007 under high biogenic amine conditions.

Overall, LPXHQ-007 may be of interest as a candidate probiotic strain, and its tyramine degradation mechanism should be interpreted as a genome-based predictive hypothesis rather than an experimentally validated pathway, which requires further biochemical and molecular validation.

### 3.6. Strain Safety Analysis

#### 3.6.1. Antibiotic Tolerance Analysis

The MIC of LPXHQ-007 against ampicillin, amoxicillin, chloramphenicol, erythromycin, tetracycline, streptomycin, kanamycin, vancomycin, norfloxacin, and gentamicin were determined using the microdilution method; see Table 3 for details. The MIC values for ampicillin, chloramphenicol, erythromycin, and tetracycline were all equal to or lower than the critical thresholds recommended by the EFSA [24], indicating that LPXHQ-007 is sensitive to these antibiotics.

Antibiotic resistance-related genes in the LPXHQ-007 genome were annotated using the CARD database. Only one predicted annotation associated with bacitracin resistance showed sequence identity close to the filtering threshold (79.6%), whereas all other hits exhibited low sequence identity (<50%). Accordingly, these matches were classified as low-confidence or putative annotations rather than confirmed antibiotic resistance genes under the applied screening criteria. Further analysis revealed that these resistance-associated annotations were all localized to chromosomal regions, and no obvious mobile genetic elements (such as transposases, integrases, or insertion sequences) were detected in their flanking regions, suggesting limited evidence for potential horizontal gene transfer under the current in silico analysis framework, rather than indicating its definitive absence, and suggesting a possible association with the strain’s inherent chromosomal characteristics. Notably, no high-risk genes encoding typical drug-inactivating enzymes, such as β-lactamases or aminoglycoside-modifying enzymes, were detected in LPXHQ-007.

The genomic localization of these resistance-associated annotations further supports their classification as intrinsic rather than acquired resistance determinants. Previous studies have suggested that intrinsic resistance traits in lactic acid bacteria are commonly chromosomally encoded and generally exhibit low transferability compared with acquired resistance genes associated with plasmids, transposons, integrative conjugative elements, or insertion sequences [25,26]. In this study, no plasmid-associated resistance regions, prophage-related loci, or obvious mobile genetic elements were detected in the flanking regions of the annotated antibiotic resistance-related genes. This provides limited in silico evidence suggesting a potentially reduced likelihood of horizontal gene transfer under the current analytical framework. These findings are generally consistent with safety evaluation principles for probiotic and food-grade lactic acid bacteria, in which the absence of identifiable transferable antibiotic resistance determinants is considered an important biosafety criterion [27]. However, definitive assessment of gene transfer potential requires more comprehensive genomic and experimental validation.

#### 3.6.2. Analysis of Virulence Factors and Harmful Metabolites

LAB are widely recognized as safe probiotics; however, some strains may carry potential virulence factors such as hemolysins, biogenic amine synthesis genes, or antibiotic resistance genes. Therefore, a systematic assessment of virulence factors is required prior to their application [28]. Based on predictions from the Virulence Factor Database (VFDB), 110 potential virulence factor genes were annotated in the LPXHQ-007 genome, the vast majority of which belong to non-classical virulence factors or possess dual functions, and generally exhibit low sequence homology (<60%). This genome does not encode genes related to hemolysin BL, cytotoxin K, enterotoxin NHE, non-hemolytic enterotoxin T, or emetotoxin.

Furthermore, annotation analysis based on the KEGG and COG databases (Table 4) indicates that LPXHQ-007 lacks genes encoding tyrosine decarboxylase (*tyrDC*), histidine decarboxylase (*hdcA*), ornithine decarboxylase (*speC*, *speF*), arginine decarboxylase (*speA*), and lysine decarboxylase (*cadA*, *ldcC*), and only two auxiliary genes were annotated. Due to the absence of a complete biosynthetic system, LPXHQ-007 cannot produce the corresponding biogenic amines.

**Figure 7:**
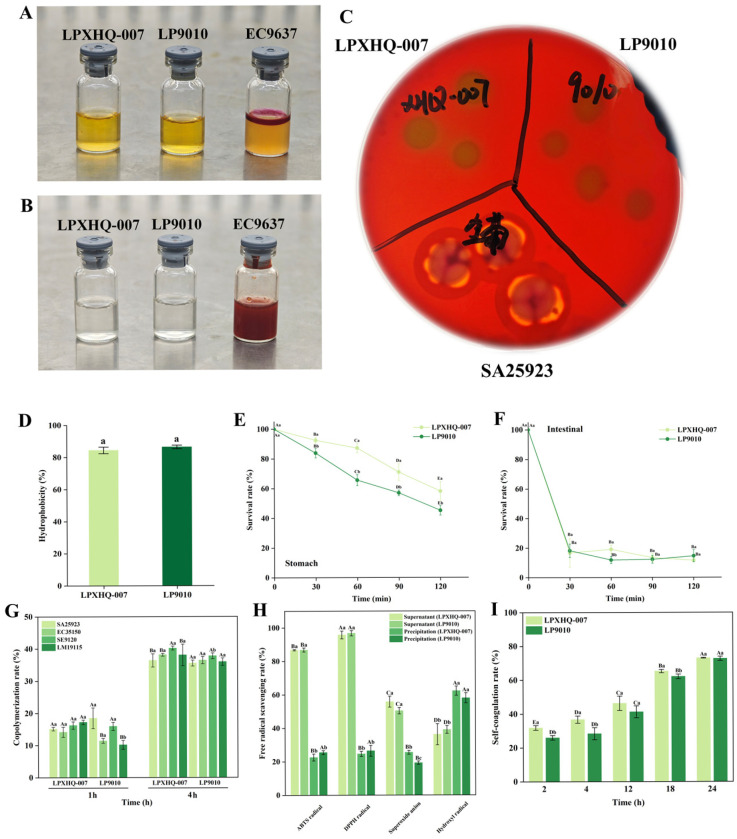
Safety and tolerance and adhesion-related analysis of LPXHQ-007. (**A**) The indole test result, (**B**) the nitrite reductase test result, and (**C**) the hemolysis test result. (**D**) is the cell surface hydrophobicity; (**E**) the tolerance to artificial gastric fluid; (**F**) the tolerance to artificial intestinal fluid; (**G**) the determination of copolymer force; (**H**) the antioxidant activity; (**I**) the assessment of self-polymerization force. Data are presented as the mean ± standard deviation (error bars). Different letters (a, b; A, B, C) marked above the bars or near data points indicate statistically significant differences as defined below: Different lowercase letters (a, b, c) indicate significant differences between the experimental group (LPXHQ-007) and the control group (LP9010) under the same condition (*p* < 0.05). Different uppercase letters (A, B, C, D, E) indicate significant differences among different treatment conditions or time points (*p* < 0.05).

Hemolytic activity is a key indicator for assessing the safety of probiotics, as it reflects a strain’s ability to destroy red blood cells. As shown in Figure 7C, after 36 h of incubation, the positive control strain SE25923 produced a distinct β-hemolytic zone on the culture medium, whereas LPXHQ-007 did not form any hemolytic zone, indicating γ-hemolytic activity. Nitrate reductase activity and indole production capacity are important indicators of a strain’s metabolic characteristics; nitrate reductase may catalyze the production of nitrites, which pose food safety risks, while indole, as a metabolite of tryptophan, is associated with interactions with the gut microbiota. The test results showed that LPXHQ-007 was negative in both the indole test (Figure 7A) and the nitrate reductase test (Figure 7B), indicating that it lacks tryptophanase and nitrate reductase activity.

### 3.7. Analysis of Probiotic Properties of Strains

#### 3.7.1. Analysis of Tolerance to Artificial Gastrointestinal Fluids

The tolerance of LPXHQ-007 was evaluated using an in vitro model simulating gastrointestinal stress. In artificial gastric fluid (pH 3.0), the survival rates of both strains decreased with increasing treatment time; however, LPXHQ-007 exhibited significantly greater acid resistance (*p* < 0.05). After 120 min of treatment, its survival rate remained at approximately 58%, whereas that of the control strain LP9010 dropped to approximately 45% (Figure 7E). In simulated intestinal fluid containing 0.3% bile salts, the survival rates of both strains dropped sharply within the initial 30 min due to the rapid destruction of cell membranes by bile salts, after which the curve flattened. During the entire treatment period, the survival rate of LPXHQ-007 was comparable to that of the control strain (Figure 7F). The results indicated that LPXHQ-007 had a superior potential for gastric tolerance compared to the control strain.

#### 3.7.2. Antioxidant Activity Analysis

The scavenging capacities of LPXHQ-007 and 9010 against hydroxyl radicals, superoxide anions, ABTS^+^, and DPPH radicals were evaluated (Figure 7H). The results showed that, except for hydroxyl radicals, the antioxidant activity of the cell-free supernatants from both strains was significantly higher than that of their bacterial suspensions. Specifically, the supernatant of LPXHQ-007 exhibited scavenging rates of 86.7%, 95.7%, and 55.8% for ABTS^+^, DPPH, and O_2_•^−^, respectively, while its bacterial suspension also achieved scavenging rates exceeding 20% for each radical. Notably, for hydroxyl radicals, the scavenging rate of the LPXHQ-007 bacterial suspension (62.2%) was higher than that of its supernatant (36.2%). In summary, LPXHQ-007 exhibits strong antioxidant capacity, which may contribute to enhanced health benefits.

#### 3.7.3. Annotation of Stress Tolerance-Related Genes

To evaluate the strain’s environmental adaptability, an annotation analysis was conducted on stress-tolerance-related genes in the LPXHQ-007 genome. The results indicate that this strain possesses a rich array of stress-response genes capable of effectively coping with temperature, pH, bile salts, oxidative, and osmotic stresses (Table 5).

LPXHQ-007 encodes a complete heat shock response system, including the *DnaK-DnaJ-GrpE* complex and the *GroES-GroEL* chaperone system—which bind to and repair heat-damaged proteins to prevent their aggregation—as well as the transcriptional repressor HrcA, which regulates the expression of these heat shock genes. In addition, its genome is annotated with various ATP-dependent proteases, including *ClpB*, *ClpC*, *ClpE*, *ClpL*, *ClpX*, *ClpP*, as well as HslUV and Lon. Together with these proteases, these chaperones form an efficient protein quality control network responsible for degrading misfolded or damaged proteins, which is essential for maintaining cellular homeostasis under stress conditions [29].

LPXHQ-007 also contains three cold shock protein (*CspA*) encoding genes. *CspA* acts as an RNA chaperone, preventing mRNA from forming secondary structures at low temperatures by binding to it. This maintains ribosome binding and normal protein translation, ensuring the strain’s survival and metabolic activity in cold environments [30].

To colonize the gastrointestinal tract, probiotics must tolerate stomach acid and bile salts. Regarding acid tolerance, LPXHQ-007 possesses a complete F_0_F_1_-ATPase system (encoding 8 F_0_F_1_-ATPases and 4 Na^+^/H^+^ antiporters). The former expels protons from the cell by consuming ATP, serving as the core mechanism for maintaining intracellular pH balance; the latter utilizes transmembrane ion gradients to regulate intracellular pH and sodium ion concentration [31]. Regarding bile salt tolerance, although no bile salt hydrolase genes were identified in its genome, two active ABC multidrug efflux systems were annotated, which can directly expel bile salts. Furthermore, the strain contains two cyclopropane fatty acid synthases, which enhance membrane rigidity and hydrophobicity by catalyzing the formation of cyclopropane rings from unsaturated fatty acid chains, thereby reducing membrane fluidity and effectively preventing bile salts from inserting into the cell membrane [32].

Furthermore, LPXHQ-007 possesses a comprehensive antioxidant system. Annotated genes include catalase (CAT), which directly and efficiently decomposes the toxic molecule hydrogen peroxide (H_2_O_2_) into water and oxygen. NADH oxidase (*nox2*) consumes oxygen to produce water, reducing intracellular oxygen partial pressure and thereby indirectly lowering ROS generation; NADH peroxidase (*Npr*) directly utilizes H_2_O_2_ to oxidize NADH [19]. Additionally, the LPXHQ-007 genome contains a complete glutathione and thioredoxin reduction system. The glutathione system comprises one glutathione peroxidase (*Gpx*) and four glutathione reductases (*Gor*). *Gpx* utilizes reduced glutathione (GSH) to reduce peroxides (such as H_2_O_2_ and organic peroxides) into harmless alcohols and water, while oxidizing itself into glutathione disulfide (GSSG). Gor then uses NADPH to reduce GSSG back to GSH, sustaining the cycle [33]. The thioredoxin system comprises four thioredoxins (*TrxA*) and one thioredoxin reductase (*TrxB*). Functionally analogous to the glutathione system, it represents an independent redox relay pathway that efficiently reduces peroxides and maintains intracellular disulfide bond balance in proteins. Notably, the presence of multiple manganese ion transporters in the genome suggests that this strain may employ a “manganese-based antioxidant” strategy. This involves accumulating Mn^2+^ to form manganese-protein complexes with superoxide dismutase-like activity to scavenge superoxide anions, thereby replacing iron-dependent enzymes and reducing the highly destructive hydroxyl radicals generated by the Fenton reaction at their source [34].

#### 3.7.4. Adhesion Analysis

Hydrophobicity, self-aggregation, and the ability to co-aggregate with pathogenic bacteria are the key physicochemical mechanisms underlying bacterial adhesion to host intestinal epithelial cells, biofilm formation, and competitive exclusion. Sortase A (*SrtA*) was identified in the LPXHQ-007 genome; this enzyme specifically recognizes and anchors proteins containing the LPXTG motif to the cell wall peptidoglycan. These surface proteins (such as mucin-binding proteins (MUBs)) can directly mediate bacterial attachment to the intestinal mucosal layer [35]. Additionally, LPXHQ-007 contains various intracellular metabolic enzymes that also function as adhesion molecules, such as enolases (*Eno*) that bind to enteropectin and plasminogen, glyceraldehyde-3-phosphate dehydrogenase (*GapA*), which can bind to human intestinal mucin proteins and fibronectin; and elongation factor Tu (*Tuf*), which acts as an adhesin to bind to host components such as fibronectin and mucins [36]. Through synergistic action, these adhesion-related factors enhance the strain’s ability to colonize the intestine by interacting with host cells, demonstrating the efficacy of LPXHQ-007 as a probiotic candidate with good intestinal adhesion potential.

In the cell surface hydrophobicity test, the hydrophobicity of LPXHQ-007 was comparable to that of the control strain 9010, being 84.44% and 86.47%, respectively (Figure 7D).

Self-coagulation ability is a key characteristic for bacterial strains to form microcolonies in the gut. As shown in Figure 7I, during the early stages of cultivation (2 h and 4 h), the self- coagulation rate of LPXHQ-007 was significantly higher than that of the control strain 9010. After 4 h of cultivation, the self- coagulation rate of LPXHQ-007 reached approximately 36.67%, demonstrating stronger self-coagulation and potential biofilm-forming capabilities, which may help enhance its colonization tolerance in the dynamic intestinal environment.

Co-agglutination capacity with pathogens is a key indicator for assessing probiotics’ ability to eliminate pathogens through physical aggregation and mitigate infection risks. In this experiment, LPXHQ-007 exhibited co-agglutination rates ranging from 36.40% to 40.21% against four common foodborne pathogens after 4 h of exposure (Figure 7G), showing no significant difference compared to strain 9010 (*p* < 0.05).

#### 3.7.5. Analysis of Antibacterial Effects

The cell-free supernatant of LPXHQ-007 exhibited antimicrobial activity against various foodborne pathogens, with the strongest inhibitory effect observed against *E. coli*, producing an inhibition zone diameter of 10.8 mm. It also demonstrated significant inhibitory effects against *S. aureus*, *L. monocytogenes*, and *Salmonella*, and its antimicrobial efficacy was superior to that of the bacterial suspension (Table 6). However, in the absence of supernatant neutralisation assays and protease treatment experiments, the specific contribution of bacteriocin-like compounds cannot be confirmed. The observed antimicrobial activity may be substantially or entirely attributed to organic acid production and the resulting pH reduction during fermentation.

AntiSMASH 7.0 analysis of LPXHQ-007 genome predicted five antimicrobial bio-synthetic gene clusters: Ripp-like, terpenoid precursors, T3PKS, terpenoids, and lac-tone-autoinducers (Figure 6A). The Ripp (ribosomal peptide synthesis and post-translational modification) cluster, associated with bacteriocin biosynthesis, represents a genomic feature indicating biosynthetic potential [37]. Key ABC transporter genes in this cluster are predicted to be involved in peptide processing and secretion [38]. However, these predictions reflect genomic potential only and do not demonstrate functional involvement in the observed inhibition zones.

Second, two terpenoid biosynthetic gene clusters were identified in LPXHQ-007. The Terpene-precursor cluster contains polyprenyl synthetase, methionine aminopeptidase and ABC transporter proteins. The more complex terpene cluster includes a hydrogen peroxide-sensitive repressor, GCN5-related N-acetyltransferase, and pyridine nucleotide-disulfide oxidoreductase. These terpenoids may contribute to general stress adaptation or membrane-related physiological functions, rather than direct antimicrobial effects, and their role in antimicrobial activity remains speculative [39]. The redox enzymes suggest potential involvement in intracellular redox balance, which may indirectly influence microbial interactions under oxidative conditions. Additionally, five *ohrR* genes (*MarR* family transcription regulators) were identified, which are associated with organic peroxide stress response rather than direct antimicrobial function.

The fourth potential biosynthetic gene cluster is associated with type III polyketases (PKS). Unlike the common type I PKS, T3PKS are structurally simple but functionally diverse. Notably, the polyisopentenol–monophosphomannose synthase identified in this gene cluster suggests that LPXHQ-007 may be involved in the synthesis of complex glycolipids or cell wall-related components [40].

In addition, LPXHQ-007 carries a lactone autoinducer gene cluster capable of producing lactone autoinducers, a key quorum sensing (QS) signal in Gram-positive bacteria [41]. This system may regulate gene expression in a cell-density dependent manner [42]. Pyrimidine nucleotide disulfide oxidoreductases within this gene cluster further link quorum sensing to energy metabolism and redox balance. Furthermore, we found that LPXHQ-007 encodes five *poxL* genes (pyruvate oxidase), four *nox2* genes (NADH oxidase), and a pair of *blpA-blpB* bacteriocins. However, these findings only indicate genomic potential for antimicrobial-related functions, rather than confirming their active role in the observed inhibition zones.

Additionally, BAGEL4 analysis predicted a plantaricin (*PLN*) gene cluster (Figure 6B). This cluster encodes regulatory and structural components typically associated with bacteriocin systems, including *plnB*, *plnC*, *plnD*, and structural genes *plnM–plnO–plnI* [43]. However, in the absence of functional assays, this cluster should be considered a putative genomic feature rather than an active determinant of antimicrobial activity. Transport and immunity genes (*plnG/plnH*) similarly reflect potential biosynthetic capacity rather than confirmed function [44].

These genomic analyses indicate potential biosynthetic capacity in LPXHQ-007, but do not demonstrate functional involvement in its antimicrobial activity. Overall, the antimicrobial effect observed in this study is most likely driven by organic acid production and associated pH reduction, while the contribution of bacteriocin-like compounds remains unverified. Further experimental validation is required to clarify these mechanisms.

## 4. Limitations and Future Prospects

Several important scientific gaps should be acknowledged in the present study. First, although genome annotation suggested potential involvement of the *cueO* system and *hpa*-related genes in tyramine metabolism, these findings remain predictive and were not supported by direct functional evidence such as enzyme activity assays, transcriptional analysis, or gene-level validation. Therefore, the proposed tyramine degradation pathway should be interpreted as a genome-guided hypothesis rather than a confirmed metabolic mechanism. Second, although abiotic incubation, heat-killed cell, and adsorption controls were included, pH-matched abiotic controls and matrix-effect evaluations were not performed. Consequently, the observed reduction in detectable tyramine under fermentation conditions cannot yet be definitively separated from possible chemical or acid-mediated effects. Third, while antimicrobial activity was observed in the cell-free supernatant and multiple bacteriocin-associated gene clusters were identified through genome mining, neutralisation, protease, catalase, and heat-treatment assays were not conducted. Therefore, the contribution of bacteriocin-like compounds to the observed inhibition phenotype remains unresolved, and the antimicrobial effect may be largely attributable to organic acid production and associated pH reduction. Finally, the probiotic potential of LPXHQ-007 was evaluated primarily through in vitro assays. Its in vivo colonization ability, physiological stability, and performance in real fermented food systems remain to be further evaluated.

## 5. Conclusions

This study integrates genomic and phenotypic analyses to characterize LPXHQ-007 as a strain with detectable tyramine reduction capacity and tolerance to acidic and bile stress conditions. Genome-wide analysis identified genes potentially associated with biogenic amine metabolism, including a multicopper oxidase (*cueO*) and a putative *hpa* gene cluster; however, these annotations should be interpreted as predictive hypotheses rather than experimentally validated functional pathways, and the exact mechanisms underlying tyramine reduction remain unresolved. Safety assessment indicated the absence of hemolytic activity and mobile antibiotic resistance determinants, suggesting a generally safe genomic profile under the conditions tested. Overall, this study provides genome-informed and phenotype-supported observations of strain LPXHQ-007 in a fermentation context. However, its potential applications in food systems and the specific biochemical basis of tyramine reduction require further experimental validation.

## Figures and Tables

**Table 1:** Average nucleotide identity (ANI) comparison between LPXHQ-007 and closely related *Lactiplantibacillus* species.

Strain	ANI (%)	Alignment Coverage (%)
*Lactiplantibacillus pentosus* A22	79.90	75.8
*Lactiplantibacillus plantarum* AR171	99.22	92.5
*Lactiplantibacillus paraplantarum* RX-8	86.44	85.2

**Table 2:** Summary of the key physiological and biochemical indicators of *L. plantarum* XHQ-007.

Project	Result	Project	Result
Catalase	-	Mannitol	+
VP	-	Aesculus chinensis	-
MR	+	Sorbitol	+
H_2_S	-	Sucrose	+
Gelatin	-	Rhamnose	+
Starch Hydrolysis	-	Maltose	+

“+” indicates a positive result, and “-” indicates a negative result.

**Table 3:** The MIC and antibiotic resistance evaluation of LPXHQ-007.

Antibiotic	MIC (mg/L)	Cut-Off Values (mg/L)	Resistance Evaluation
Ampicillin	1	2	S
Amoxicillin	2	n.r.	-
Chloramphenicol	8	8	S
Erythromycin	1	1	S
Tetracycline	16	32	S
Streptomycin	128	64	R
Kanamycin	128	64	R
Vancomycin	128	n.r.	-
Norfloxacin	32	n.r.	-
Gentamicin	32	16	R

Note: S stands for sensitive, R stands for resistant, n.r. stands for not reported. Antibiotic cut-off values refer to the criteria issued by EFSA.

**Table 4:** Genes associated with the biogenic amine biosynthesis in *L. plantarum* XHQ-007.

Biogenic Amine Type	Gene Name	Gene Function	Gene ID
Tyramine	*tyrDC*	Tyrosine decarboxylase	ND
	*tyrS*	Tyrosyl-tRNA synthetase	ND
	*tyrP*	Putative tyrosine/tyramine permease	ND
	*nhaC*	Na^+^/H^+^ antiporter	GL000169
Histamine	*hdcA*	Histidine decarboxylase	ND
	*hdcB*	Active decarboxylase	ND
	*hdcP*	Histidine/histamine antiporter	ND
	*hisS*	Histidyl-tRNA synthetase	GL001716
Putrescine	*speA*	Arginine decarboxylase	ND
	*speC*	Ornithine decarboxylase	ND
	*speF*	Ornithine decarboxylase	ND
	*potE*	Transmembrane substrate/product exchanger protein	ND
Agmatine	*aguA*	Agmatine deiminase	ND
	*aguB*	Putrescine transcarbamylase	ND
	*aguC*	Specific carbamate kinase	ND
	*aguD*	Agmatine/putrescine antiporter	ND
Cadaverine	*cadA*, *ldcC*	Lysine decarboxylase	ND
	*cadB*	Cadaverine/lysine antiporter	ND
	*cadC*	Transcriptional activator of cad operon	ND

Note: ND indicates that no related gene was retrieved in the genome of LPXHQ-007.

**Table 5:** Analysis of tolerance gene of LPXHQ-007.

Gene ID	Gene Name	Function
**Universal stress family protein**		
GL001922	*GSP13*	general stress protein 13
**Proteases and chaperones**		
GL003029	*clpL*	ATP-dependent Clp protease ATP-binding subunit ClpL
GL000806	*clpC*	ATP-dependent Clp protease ATP-binding subunit ClpC
GL000605	*clpP*, *CLPP*	ATP-dependent Clp protease, protease subunit
GL001055	*clpE*	ATP-dependent Clp protease ATP-binding subunit ClpE
GL001156	*clpP*, *CLPP*	ATP-dependent Clp protease, protease subunit
GL001600	*hslU*	ATP-dependent HslUV protease ATP-binding subunit HslU
GL001601	*hslV*, *clpQ*	ATP-dependent HslUV protease, peptidase subunit HslV
GL001647	*clpB*	ATP-dependent Clp protease ATP-binding subunit ClpB
GL001822	*clpX*, *CLPX*	ATP-dependent Clp protease ATP-binding subunit ClpX
GL001836	——	Lon-like protease
**Heat-shock stress**		
GL001742	*hrcA*	heat-inducible transcriptional repressor
GL001739	*dnaJ*	molecular chaperone DnaJ
GL001740	*dnaK*, *HSPA9*	molecular chaperone DnaK
GL001741	*GRPE*	molecular chaperone GrpE
GL000450	*hslR*	ribosome-associated heat shock protein Hsp15
GL000456	*hslO*	molecular chaperone Hsp33
GL000554	*groES*, *HSPE1*	chaperonin GroES
GL000555	*groEL*, *HSPD1*	chaperonin GroEL
GL002299	*HSP20*	HSP20 family protein
GL002860	*HSP20*	HSP20 family protein
GL000116	*HSP20*	HSP20 family protein
**Cold-shock stress**		
GL000030	*cspA*	cold shock protein
GL000790	*cspA*	cold shock protein
GL000980	*cspA*	cold shock protein
**Bile salt stress resistance**		
GL001468	*cfa*	cyclopropane-fatty-acyl-phospholipid synthase
GL002718	*cfa*	cyclopropane-fatty-acyl-phospholipid synthase
**Acid stress**		
GL002848	*nhaC*	Na+:H+ antiporter, NhaC family
GL000169	*nhaC*	Na+:H+ antiporter, NhaC family
GL000350	*napA*, *nhaS3*, *nhaS5*, *gerN*	Na+:H+ antiporter
GL002435	*napA*, *nhaS3*, *nhaS5*, *gerN*	Na+:H+ antiporter
GL002042	*ATPF1E*, *atpC*	F-type H+-transporting ATPase subunit epsilon
GL002043	*ATPF1B*, *atpD*	F-type H+/Na+-transporting ATPase subunit beta
GL002044	*ATPF1G*, *atpG*	F-type H+-transporting ATPase subunit gamma
GL002045	*ATPF1A*, *atpA*	F-type H+/Na+-transporting ATPase subunit alpha
GL002046	*ATPF1D*, *atpH*	F-type H+-transporting ATPase subunit delta
GL002047	*ATPF0B*, *atpF*	F-type H+-transporting ATPase subunit b
GL002048	*ATPF0C*, *atpE*	F-type H+-transporting ATPase subunit c
GL002049	*ATPF0A*, *atpB*	F-type H+-transporting ATPase subunit a
GL000613	*clcA*, *clcB*, *CLC-E*, *CLC-F*	chloride channel protein, CIC family
**Osmotic stress**		
GL000332	*opuC*	osmoprotectant transport system substrate-binding protein
GL000332	*opuBD*	osmoprotectant transport system permease protein
GL000333	*opuA*	osmoprotectant transport system ATP-binding protein
GL001386	*opuA*	osmoprotectant transport system ATP-binding protein
GL001387	*opuBD*	osmoprotectant transport system permease protein
GL001388	*opuC*	osmoprotectant transport system substrate-binding protein
GL001389	*opuBD*	osmoprotectant transport system permease protein
GL001491	*TC.APA*	basic amino acid/polyamine antiporter, APA family
GL002802	*TC.APA*	basic amino acid/polyamine antiporter, APA family
GL002840	*TC.BCT*	betaine/carnitine transporter, BCCT family
**Oxidative stress**		
GL001683	*nox2*	NADH oxidase (H_2_O-forming)
GL002201	*npr*	NADH peroxidase
GL002926	*nox2*	NADH oxidase (H_2_O-forming)
GL000583	*nox2*	NADH oxidase (H_2_O-forming)
GL000589	*nox2*	NADH oxidase (H_2_O-forming)
GL003023	*katE*, *CAT*, *catB*, *srpA*	catalase
GL000194	*gpx*, *btuE*, *bsaA*	glutathione peroxidase
GL000334	*GSR*, *gor*	glutathione reductase (NADPH)
GL001040	*GSR*, *gor*	glutathione reductase (NADPH)
GL001585	*GSR*, *gor*	glutathione reductase (NADPH)
GL002793	*GSR*, *gor*	glutathione reductase (NADPH)
GL000206	*TXN*, *trxA*	thioredoxin
GL000584	*trxB*, *TRR*	thioredoxin reductase (NADPH)
GL001958	*TXN*, *trxA*	thioredoxin
GL002002	*tpx*	thioredoxin-dependent peroxiredoxin
GL002270	*TXN*, *trxA*	thioredoxin
GL002917	*TXN*, *trxA*	thioredoxin
GL000240	*mntH*	manganese transport protein
GL001075	*mntH*	manganese transport protein
GL002567	*mntH*	manganese transport protein
GL000923	*mntA*	manganese transport system ATP-binding protein
GL000924	*mntB*	manganese transport system permease protein
GL000925	*mntC*	manganese transport system substrate-binding protein
**Nitrite stress**		
GL001908	*pgl*	6-phosphogluconolactonase

**Table 6:** Antibacterial effect of LPXHQ-007.

Pathogenic Bacteria	Cell-Free Supernatant (mm)	Bacterial Suspension (mm)	Cell-Free Supernatant (mm)	Bacterial Suspension (mm)
	LPXHQ-007		LP9010	
*E. coli* ATCC 35150	10.80 ± 0.43 ^a^	5.71 ± 0.20 ^a^	10.25 ± 0.34 ^a^	5.31 ± 0.32 ^a^
*S. aureus* ATCC 25923	5.72 ± 0.66 ^a^	2.31 ± 0.45 ^a^	5.52 ± 0.15 ^a^	0 ^b^
*L. monocytogenes* ATCC 19115	4.51 ± 0.14 ^a^	2.08 ± 0.31 ^a^	4.62 ± 0.18 ^a^	0 ^b^
*S. enterica* ATCC 9120	5.57 ± 0.80 ^a^	2.76 ± 0.64 ^a^	5.43 ± 0.32 ^a^	0 ^b^

Different superscripted lowercase letters in the table peers represent significant differences between the two strains (supernatant and bacterial) (*p* < 0.05).

## Data Availability

The whole-genome sequencing project for LPXHQ-007 has been deposited in the GenBank database of the National Center for Biotechnology Information (NCBI) under accession number JBXYMJ000000000, as part of BioProject PRJNA1459679. The version described in this paper is JBXYMJ010000000. The corresponding BioSample accession number is SAMN57562669, and the NCBI submission identifier is SUB16156224. The original contributions presented in this study are included in the article/Supplementary Materials. Further inquiries can be directed to the corresponding authors.

## Supplementary Materials

The following supporting information can be downloaded at: https://www.mdpi.com/article/10.3390/foods15111977/s1, Figure S1: HPLC chromatograms of tyramine degradation by the top 5 strains. A: Chromatogram of a standard tyramine solution (500 mg/L). B–F: Chromatograms of histamine after treatment with the top 5 degrading strains (strains 40, 43, 15, 37, and 46, respectively); Figure S2: Calibration curve for tyramine; Figure S3: Comparison of tyramine reduction by viable cells, heat-killed cells, and adsorption controls of strain 46. Data are presented as mean ± standard deviation (SD) from three independent experiments. Different lowercase letters indicate significant differences among groups (*p* < 0.05); Table S1: HPLC elution profile; Table S2: Tyramine degradation rate.
