# Supplementary material for: Combined Genome-Wide and Phenotypic Profiling of Lactiplantibacillus plantarum XHQ-007: Genome-Guided Insights into Tyramine Reduction, Safety Characteristics, and Probiotic Potential
Source: Foods. 2026 Jun 2;15(11):1977. doi: 10.3390/foods15111977 (PMC13256593; doi:10.3390/foods15111977)
Supplement: Supplementary file 1 [file foods-15-01977-s001.zip › foods-4318661-supplementary.pdf]

Table S1. HPLC elution profile

Time (min)	Mobile phase A (%)	Mobile phase B (%)
0	65	35
10	8	92
17	25	75
20	40	60
25	65	35

Table S2. Tyramine degradation rate

**Strains**	**Final tyramine concentration (mg/L)**			**Tyramine degradation rate (%)**
	**Parallel 1**	**Parallel 2**	**Parallel 3**	
15	178.10	184.75	120.98	67.74±7.01[a]
46	51.15	69.94	179.81	79.94±13.9[a]
37	106.74	103.53	183.63	73.74±8.26[a]
40	197.94	177.96	221.55	60.17±4.37[b]
18	194.71	257.38	285.94	50.8±9.33[b]
43	159.70	189.60	219.50	62.08±5.98[a]
21	292.92	206.85	274.68	48.37±9.07[b]
31	172.65	219.24	257.20	56.73±8.47[b]
33	306.15	262.37	238.63	46.19±6.85[c]
30	279.22	277.09	335.89	40.52±6.67[c]

Note: Data are presented as mean ± standard deviation. Different lowercase letters indicate significant differences among the samples ($p < 0.05$)

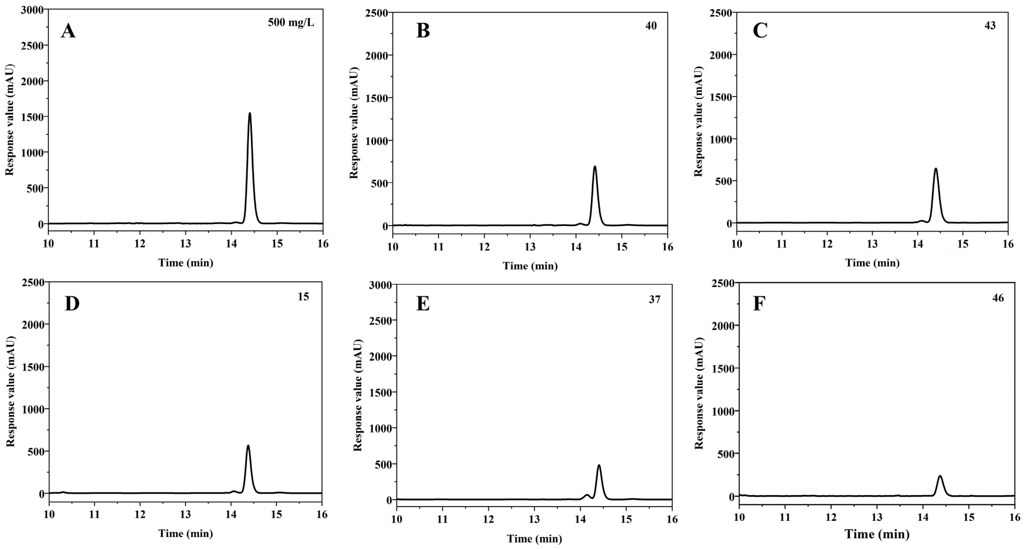

Figure S1. HPLC chromatograms of tyramine degradation by the top 5 strains. A: Chromatogram of a standard tyramine solution (500 mg/L). B–F: Chromatograms of histamine after treatment with the top 5 degrading strains (strains 40, 43, 15, 37, and 46, respectively).

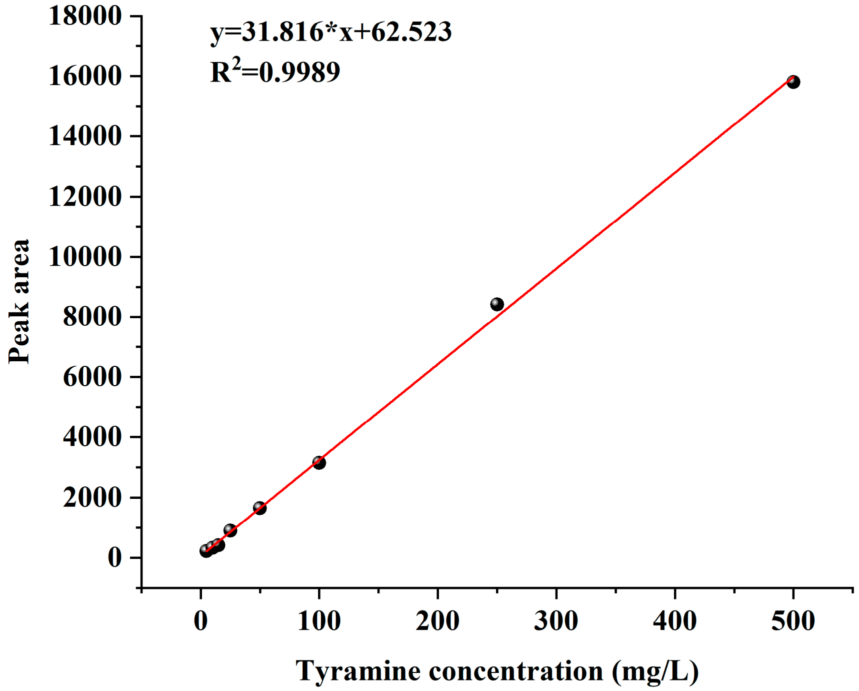

Figure S2. Calibration curve for tyramine.

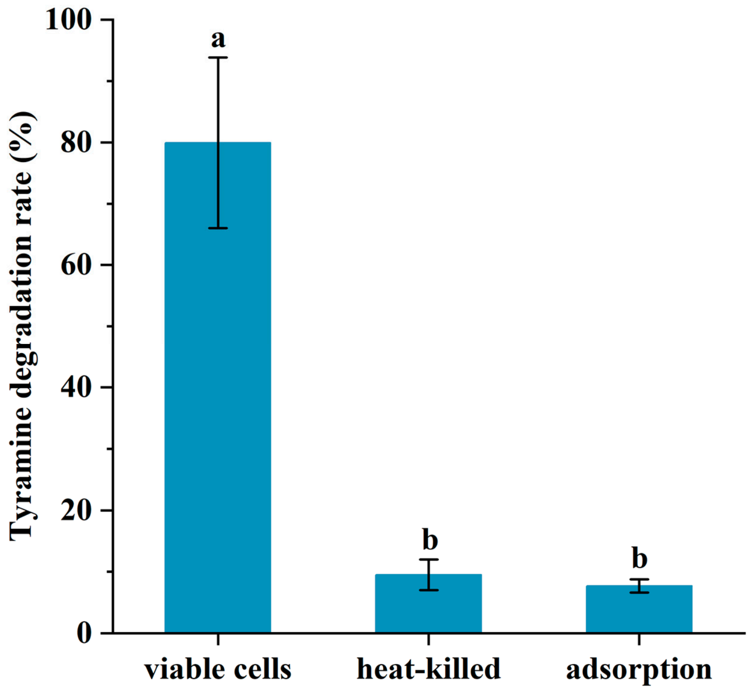

Figure S3. Comparison of tyramine reduction by viable cells, heat-killed cells, and adsorption controls of strain 46. Data are presented as mean ± standard deviation (SD) from three independent experiments. Different lowercase letters indicate significant differences among groups ($p < 0.05$).
